# Probing the nuclear import signal and nuclear transport molecular determinants of PRV ICP22

**DOI:** 10.1186/s13578-016-0069-7

**Published:** 2016-01-25

**Authors:** Mingsheng Cai, Si Jiang, Zhancheng Zeng, Xiaowei Li, Chuncong Mo, Yanjia Yang, Chunke Chen, Peiping Xie, Yun Bian, Jinlin Wang, Jinlu Huang, Daixiong Chen, Tao Peng, Meili Li

**Affiliations:** Department of Pathogenic Biology and Immunology, School of Basic Science, Guangzhou Medical University, Guangzhou, 511436 People’s Republic of China; Guangzhou Hoffmann Institute of Immunology, School of Basic Science, Guangzhou Medical University, Guangzhou, 511436 People’s Republic of China; Guangdong Haid Group Co., Ltd., Guangzhou, 511400 People’s Republic of China

**Keywords:** PRV ICP22, Nuclear transport, Nuclear localization signal (NLS), Importin, Ran-GTP

## Abstract

**Background:**

Herpes simplex virus 1 (HSV-1) ICP22 is a multifunctional protein and important for HSV-1 replication. Pseudorabies virus (PRV) ICP22 (P-ICP22) is a homologue of HSV-1 ICP22 and is reported to be able to selectively modify the transcription of different kinetic classes of PRV genes, however, the subcellular localization, localization signal and molecular determinants for its transport to execute this function is less well understood.

**Results:**

In this study, by utilizing live cells fluorescent microscopy, P-ICP22 fused to enhanced yellow fluorescent protein (EYFP) gene was transient expressed in live cells and shown to exhibit a predominantly nucleus localization in the absence of other viral proteins. By transfection of a series of P-ICP22 deletion mutants fused to EYFP, a bona fide nuclear localization signal (NLS) and its key amino acids (aa) of P-ICP22 was, for the first time, determined and mapped to aa 41–60 (PASTPTPPKRGRYVVEHPEY) and aa 49–50 (KR), respectively. Besides, the P-ICP22 was demonstrated to be targeted to the nucleus via Ran-, importin α1-, and α7-mediated pathway.

**Conclusions:**

Our findings reported herein disclose the NLS and molecular mechanism for nuclear transport of P-ICP22, these results will uncover new avenues for depicting the biological roles of P-ICP22 during PRV infection.

## Background

Pseudorabies virus (PRV), a member of genus *Varicellovirus* of the subfamily alphaherpesvirinae, is a pathogen of Aujeszky’s disease that can infect most mammals except for the higher-order primates. However, the neurotropic nature of PRV makes it a useful tool of neuronal connections and a useful model organism for the research of herpesvirus molecular pathomechanisms [1]. PRV is reported to be closely related to human pathogens varicella-zoster virus (VZV), herpes simplex virus 1 and 2 (HSV-1 and HSV-2) [2], and the animal herpesvirus bovine herpesvirus type 1 (BHV-1) [3]. To analyze the fundamental mechanisms underlying PRV spreading and pathogenesis, it is critical to gain a comprehensive understanding of the roles of each gene and their products during viral replication. In the course of lytic cycle of infection, PRV genes are expressed as other herpesviruses in a sequential cascade manner: immediate-early (IE), early (E) and late (L). PRV ICP22 (P-ICP22), the gene product of *US1*, does not express as an IE protein in the PRV life cycle, whereas its homologues HSV-1 ICP22 and VZV ORF63 are demonstrated to act as IE proteins [4], and BHV-1 BICP22 exhibits IE and L transcription kinetics [5]. As important proteins for herpesviruses replication, P-ICP22 homologues play many roles in various aspects during infection, such as BHV-1 BICP22 is documented to act as a transrepressor protein on viral promoters of different kinetic classes [6]. Moreover, HSV-1 ICP22 is implicated in ensuring proper virion morphology [7], inducing the formation of discrete nuclear foci containing cellular chaperone proteins known as VICE domains [8], modulating viral and cellular genes expressions or their activity [9, 10] and phosphorylated by viral and cellular kinases and nucleotidylylated by casein kinase II [11, 12]. Besides, VZV ORF63 is also involved in modulating viral and cellular genes expressions [13, 14], inhibiting apoptosis of primary human neurons [15] and the efficient establishment of latency [14]. Recently, a paper reported that P-ICP22 is able to selectively modify the transcription of different kinetic classes of PRV genes, this may probably happen in the nucleus and/or the cytoplasm [16], however, the subcellular localization, transport signal and nucleo-cytoplasmic transport mechanism utilized by P-ICP22 to execute this function is less well understood.

In this work, live cells fluorescence microscopy and co-immunoprecipitation (Co-IP), which are widely applied and developed in our group [17–24], were employed to characterize the exact subcellular localization, localization signal and transport mechanism of P-ICP22. We showed that P-ICP22 localized predominantly to the nucleus in the absence of other viral proteins in transient transfected live cells. By sequence analysis and constructing a series of deletion mutants of P-ICP22 fused to enhanced yellow fluorescent protein (EYFP), the precise nuclear localization signal (NLS) and its key amino acids (aa) of P-ICP22 was identified and mapped to aa 41–60 and aa 49–50, respectively. Besides, the molecular mechanisms of nuclear import, which are crucial for understanding P-ICP22-mediated biological effects in PRV infection cycle, were characterized to target to the nucleus through a Ran-, importin α1- and α7-dependent nuclear import pathway.

## Results and discussion

### Subcellular localization of P-ICP22 in the transfected live cells

**Fig. 1:**
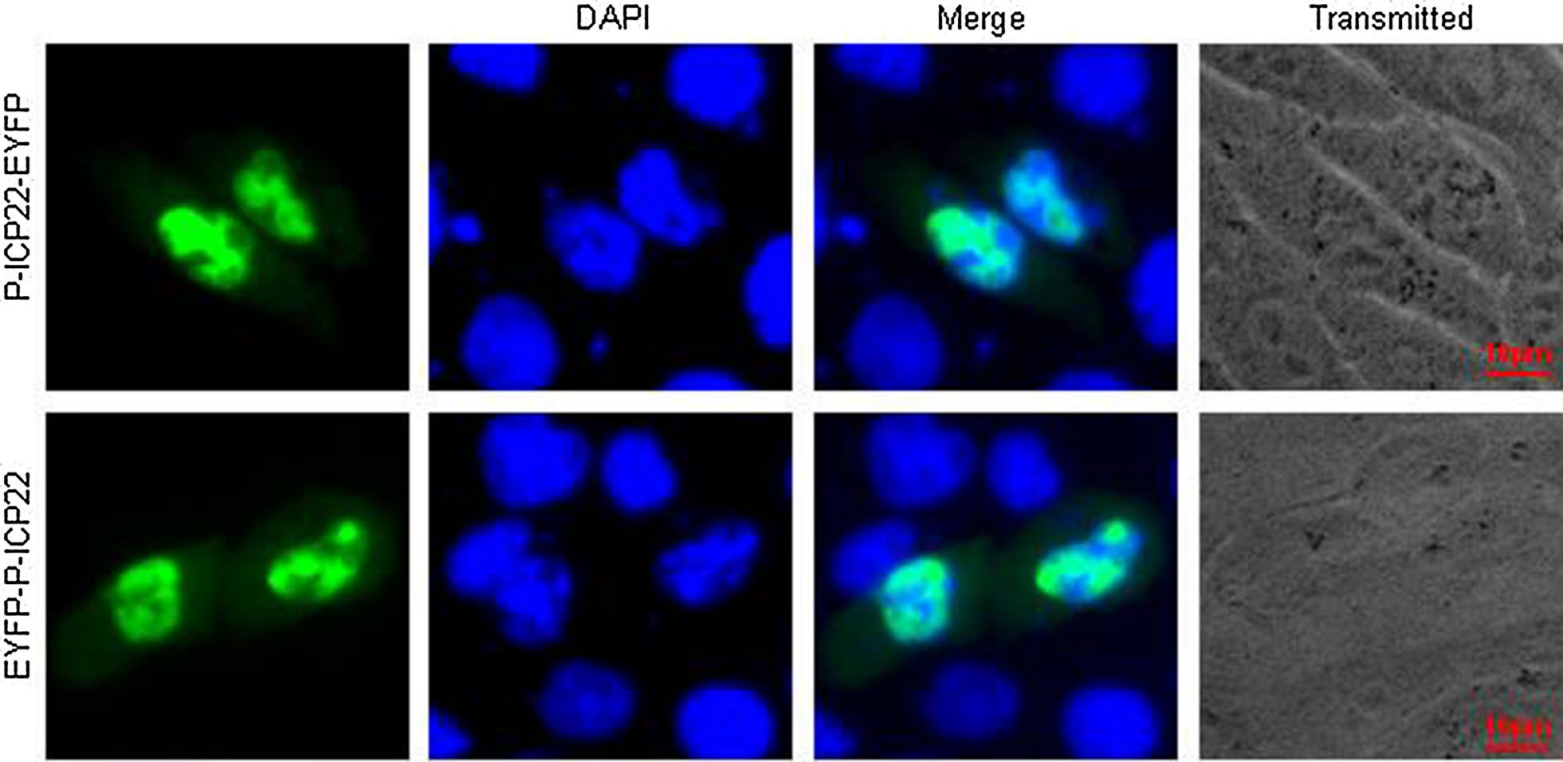
Subcellular localization of P-ICP22 in transfected living cells. Subcellular distribution of P-ICP22-EYFP and EYFP-P-ICP22 in transfected-COS-7 cells. Cells were stained with DAPI to visualize the nuclei. All *scale bars* indicate 10 um

It is common knowledge that verification of subcellular localization is one way to disclose the potential roles of some proteins. In the present study, the fate of P-ICP22 distribution was determined by fluorescence microscopy technique. Plasmid encoding P-ICP22 fused to the N terminus of EYFP was constructed and transfected into COS-7 cells to detect the subcellular localization of P-ICP22 in live cells in the absence of other viral proteins. As a result, P-ICP22-EYFP exhibited predominantly nuclear localization (Fig. 1), which is similar with the result that in HSV-1-infected cell ICP22 also mainly locates in the nucleus, particularly in the small, dense nuclear bodies early in infection and in the diffuse replicative complexes after the onset of DNA synthesis [25].

Although P-ICP22 was predicted to be localized mainly in the cytoplasm [26], it was substantiated here that it predominantly accumulates in nucleus, it’s well known that protein phosphorylation is one of the most normal and essential types of protein modification, and certain aspects of cell process modulation are regulated by protein phosphorylation. Signal transduction, proliferation, differentiation and metabolism are all controlled by the balance of activity of protein kinases and protein phosphatases upon pivotal target proteins. Phosphorylation site prediction revealed there is 22 potential phosphorylation sites in P-ICP22, including 15 serine, 5 threonine and 2 tyrosine residues [26]. Tyrosine phosphorylation is reported to be involved in the replication of several herpesviruses [27, 28] and in shifting protein translocation from the cytoplasm to the nucleus during productive virus infection [29]. Phosphorylation of VZV ORF63 is also associated with its subcellular localization and transcriptional regulatory properties [30, 31]. Therefore, the subcellular localization differences of P-ICP22 between the predicted result and experiment result may be due to the presence of potential phosphorylation sites especially the tyrosine residues, and P-ICP22 phosphorylation may also play an important role during PRV infection, perhaps in modulating its subcellular localization or other uncharacterized functions, such as transcriptional regulation.

In an attempt to examine whether the location of EYFP influences the localization of P-ICP22 in cells, a plasmid expressing P-ICP22 fused to the C terminus of EYFP (EYFP-P-ICP22) was constructed, and fluorescence microscopy showed identical subcellular distribution patterns of P-ICP22-EYFP with EYFP-P-ICP22 (Fig. 1). Therefore, subsequent experiments were done using pP-ICP22-EYFP.

### Characterization of the NLS and its key aa of P-ICP22

**Fig. 2:**
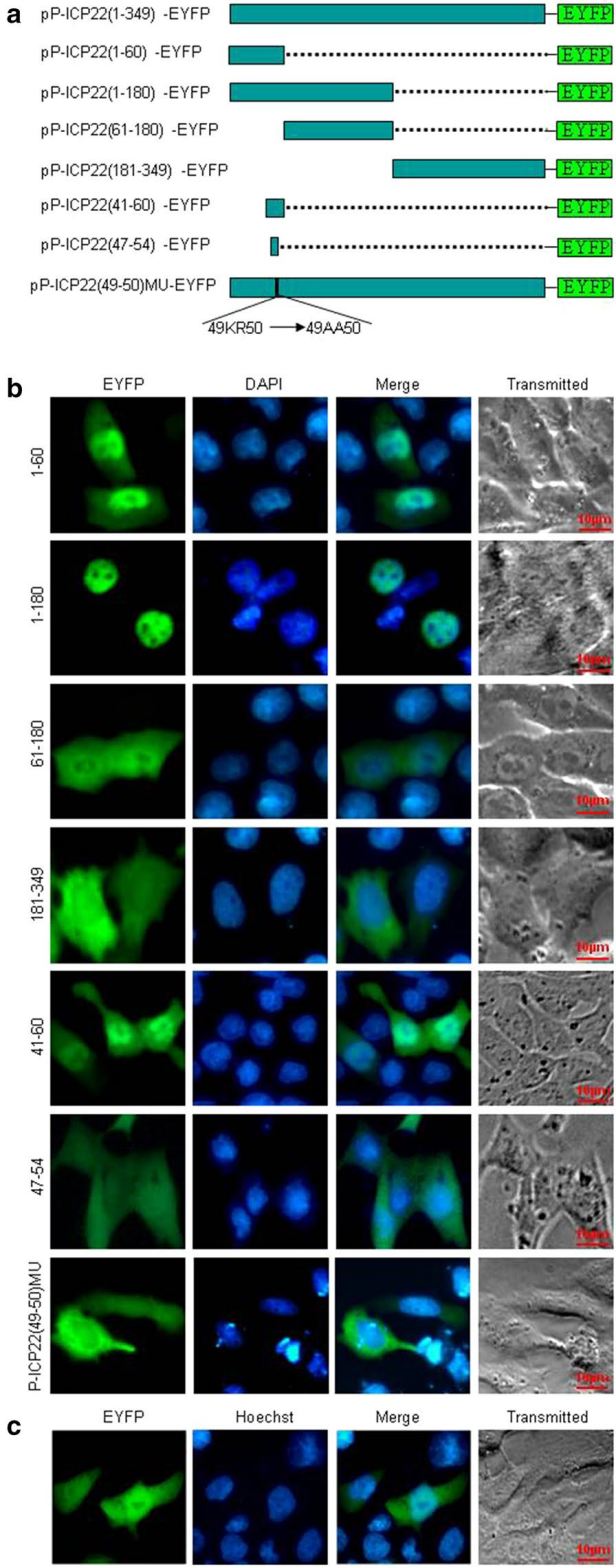
Subcellular localization of the P-ICP22 deletion mutants. **a** Schematic representation of wild-type P-ICP22 protein and its N- and C-terminal deletion mutants fused with EYFP. **b** Subcellular localization of these P-ICP22 deletion mutants shown in (**a**). **c** Subcellular distribution of the negative control EYFP in transfected-COS-7 cells. Cells were stained with DAPI to visualize the nuclei. All *scale bars* indicate 10 um

It is well known that NLSs are mainly composed of basic residues [32]. Sequence analysis using PSORT II predicted that P-ICP22 has a potential NLS in the arginine-rich region, namely PPKRGRYV at aa 47–54 (contains ^49^KR^50^). In order to determine whether this potential NLS is functional, firstly, two deletion mutants (aa1-180-EYFP and aa181-349-EYFP) were constructed within the P-ICP22-EYFP fusion protein (Fig. 2a). Then, these two constructs were tested in COS-7 cells. As shown in Fig. 2b, the fluorescence of aa1-180-EYFP showed absolutely nuclear localization, whereas the fluorescence of 181-349-EYFP showed cytoplasmic localization, suggesting aa 1–180 contains functional NLS. To further determine the functional NLS, plasmids encoding EYFP fused to two different peptides (aa 1–60 and aa 61–180) were constructed (Fig. 2a) and tested in COS-7 cells. As shown in Fig. 2b, fluorescence of aa1-60-EYFP was identical to that of aa1-349-EYFP. In contrast, fluorescence of aa61-180-EYFP showed identical subcellular localization pattern to the negative control EYFP (Fig. 2c), with diffuse fluorescence throughout both the cytoplasm and the nucleoplasm (pan-cellular subcellular localization), suggesting that the region of aa 1–60 contains a functional NLS.

To finally define the minimum region that has the ability to direct EYFP to the nucleus, plasmids encoding EYFP fused to aa 41–60 and aa 47–54 were constructed (Fig. 2a) and tested in COS-7 cells. As shown in Fig. 2b, fluorescence of aa47-54-EYFP was identical to that of EYFP, whereas fluorescence of aa41-60-EYFP showed predominant nuclear localization, suggesting the predicted NLS (^47^PPKRGRYV^54^) is not a genuine NLS, and the functional NLS in P-ICP22 is a 20-residue peptide ^41^PASTPTPPKRGRYVVEHPEY^60^, and its key aa may be ^49^KR^50^.

To further verify this deduction, basic residues within the ^49^KR^50^ sequences of P-ICP22 was mutated with neutral alanine residues to yield ^49^AA^50^, and fused with EYFP to create pP-ICP22-49-50mut-EYFP (Fig. 2a). As shown in Fig. 2b, mutation of aa 49–50 within P-ICP22 abrogated the nuclear localization of P-ICP22, showing pan-cytoplasm subcellular localization pattern, confirming aa 49–50 is the key aa of NLS for the nuclear localization of P-ICP22.

### Characterization of the nuclear import mechanisms of P-ICP22

**Fig. 3:**
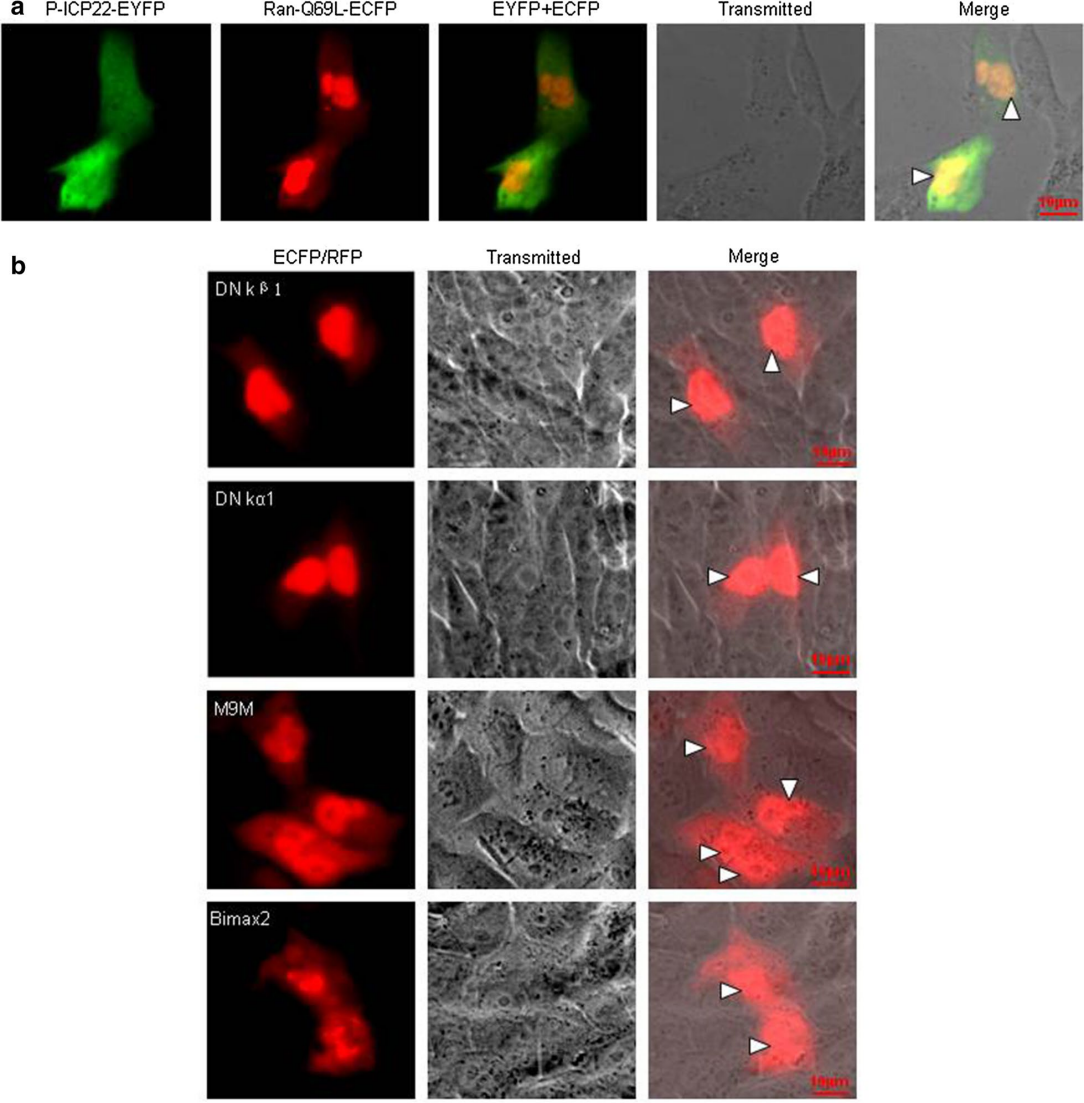
Ran-dependent nuclear import of P-ICP22. **a** Fluorescence microscopy of COS-7 cells co-transfected with plasmids pP-ICP22-EYFP and pRan-Q69L-ECFP. **b** Fluorescence microscopy of COS-7 cells only transfected with plasmid encoding DN kβ1-ECFP, DN kα1-ECFP, M9M-RFP or Bimax2-RFP. The *white arrowhead* showed the nuclei in a cell that co-transfected with two plasmids. All *scale bars* indicate 10 um

The Ran protein has been documented to be required for NLS dependent nuclear import, and most transport processes studied to date require the Ran GTPase protein [33]. In order to investigate the nuclear import mechanism of P-ICP22, a DN RanGTP (Ran-Q69L), which lacks the ability in GTP hydrolysis [34], was used to probe whether Ran is required for the nuclear transport of P-ICP22. COS-7 cells were co-transfected with P-ICP22-EYFP and Ran-Q69L-ECFP, and their subcellular localization patterns were observed. As a result, co-transfection of Ran-Q69L significantly blocked the nuclear import of P-ICP22 (Fig. 3a), indicating that P-ICP22 protein is a Ran-dependent protein and is transported into the nucleus from the cytoplasm through a classical nuclear transport pathway.

**Fig. 4:**
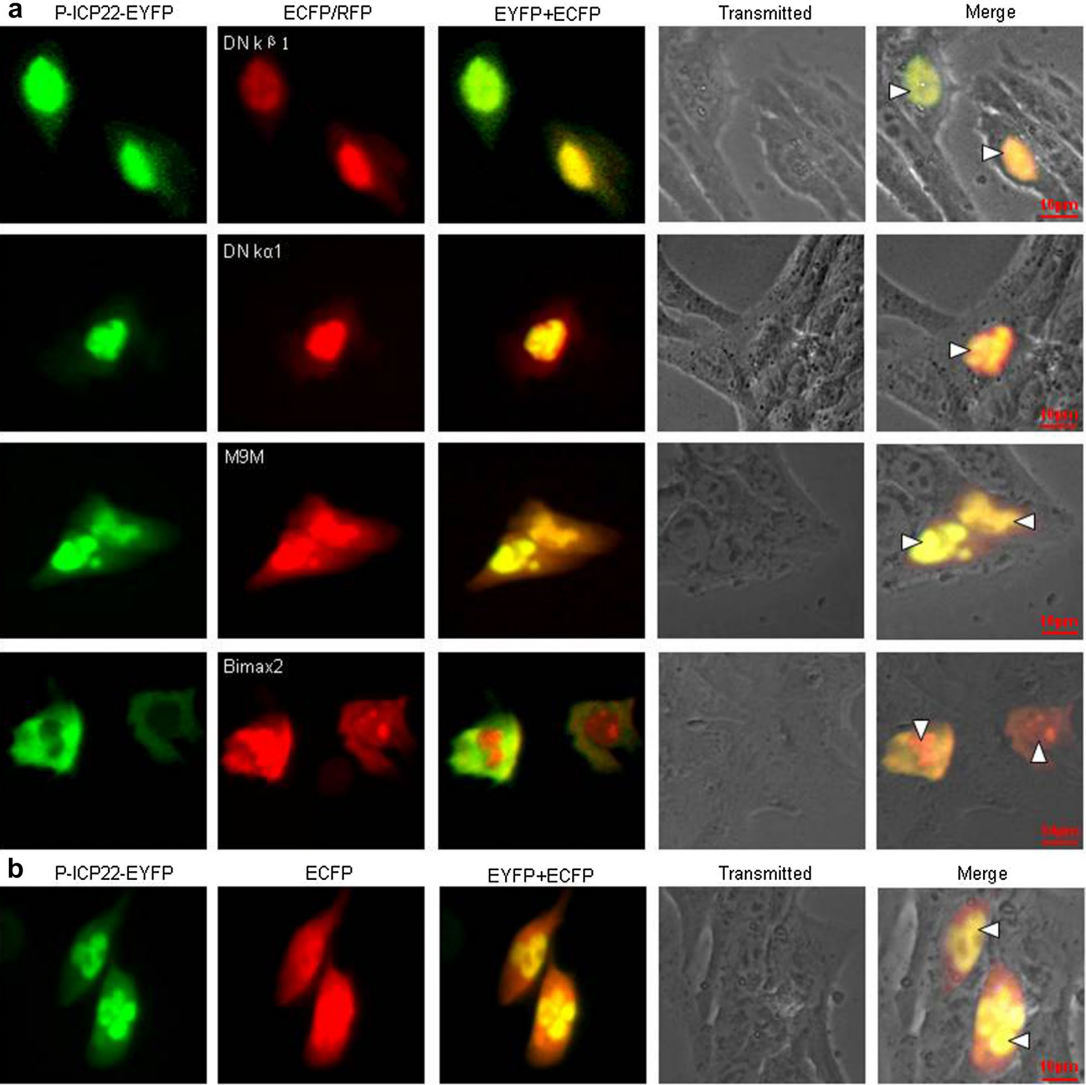
The nuclear import mechanism of P-ICP22. **a** Fluorescence microscopy of COS-7 cells co-transfected with plasmid pP-ICP22-EYFP and plasmid encoding DN kβ1-ECFP, DN kα1-ECFP, M9M-RFP or Bimax2-RFP, respectively. **b** Fluorescence microscopy of COS-7 cells co-transfected with pP-ICP22-EYFP and pECFP-N1. The *white arrowhead* showed the nuclei in a cell that co-transfected with two plasmids. All *scale bars* indicate 10 um

A well-characterized NLS is recognized by members of the importin family of cellular transporters. As documented, the importin α/β heterodimer, where importin α recognizes the NLS, and importin β promotes the importin α-NLS binding by modulating a conformational change in importin α [35]. Then, the importin α/β-cargo complex is imported to the nucleus and is separated by the Ran-GTP binding with importin β1 [36]. In mammals, there are at least six isoforms of karyopherins-α, which could be calssified into three different subfamilies on the basis of their sequence similarity: the Rch1 subfamily (importin α1/Rch1), the Qip1 subfamily (importin α3/Qip1 and importin α4/Qip2), and the NPI-1 subfamily (importin α5/NPI-1, importin α6, and importin α7/NPI-2) [37, 38]. Besides the importin α/β mechanism, it is described that there are at least ten importin β-dependent nuclear import/export pathways [39], the NLSs of which could be directly recognized by several importin β members, especially the extensively studied importin β1 and importin β2 (transportin-1) [40]. To pursue the cellular receptor assume P-ICP22 nuclear targeting and further explore the nuclear import pathway of P-ICP22, DN importin β1 (DN kβ1) [41] and DN importin α5 (DN kα1) [42], which deficient in binding Ran and importin β [43, 44], respectively, and nuclear transport inhibitors M9M and Bimax2 that are specific for the transportin-1 [45] or importin α1, α3, α6 and α7 [46] pathways, respectively, were also introduced to confirm whether they are required for the nuclear transport of P-ICP22. Compared with the negative control cells that only transfected with DN kβ1, DN kα1, M9M or Bimax2 (Fig. 3b), co-transfection of the negative control ECFP (Fig. 4b), DN kβ1, DN kα1 or M9 M did not changed the localization of P-ICP22 (Fig. 4a), however, Bimax2 could obviously impair the nuclear import of P-ICP22 and redistributed it to the cytoplasm, suggesting that P-ICP22 may be transported into the nucleus by at least one of proteins from importin α1, α3, α6 or α7, but not importin β1, importin α5 or transportin-1.

### P-ICP22 binds importin α1 and importin α7

**Fig. 5:**
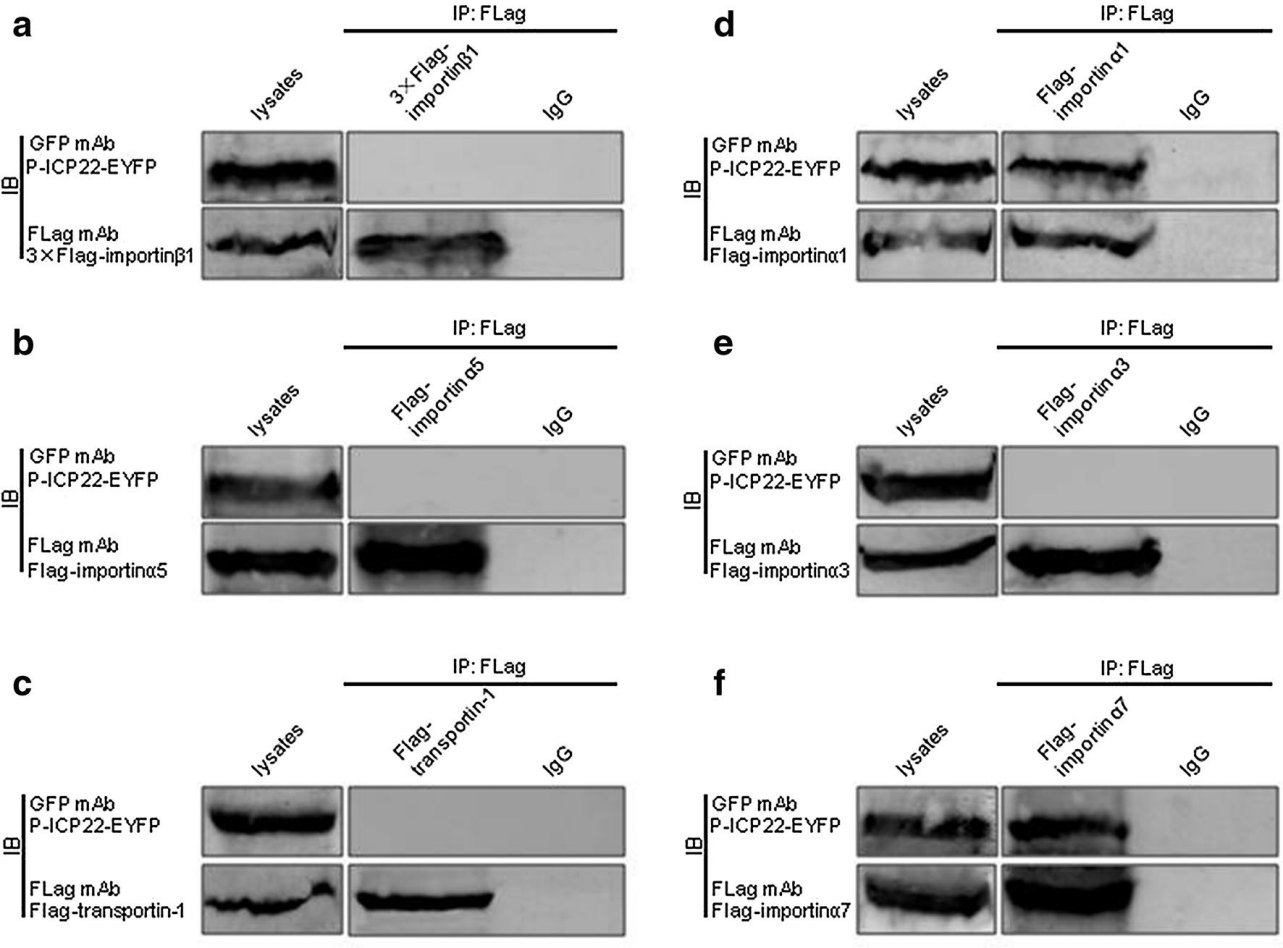
Investigation of the interactions between P-ICP22 and importin β1, transportin-1, importin α5, importin α1, importin α3 or importin α7. Co-IP of P-ICP22 with importin β1 (**a**), importin α5 (**b**), Transportin-1 (**c**), importin α1 (**d**), importin α3 (**e**) or importin α7 (**f**). HEK293T cells were co-transfected with pP-ICP22-EYFP and pCMV9-3 × Flag-importin β1 (3 × Flag-importin β1), pcDNA-Flag-kα1 (Flag-importin α5), pFLAG-CMV-transportin-1 (Flag-transportin-1), Flag-importin α1, Flag-importin α3 or Flag-importin α7. 24 h after transfection, cells were lysed and IP with anti-Flag or mouse IgG control. Immunoprecipitated proteins as well as the cell lysates were separated in denaturing 10 % SDS-PAGE, and analyzed by IB with anti-Flag mAb or anti-YFP pAb

It is well known that distinct type of cells can express almost all the importins (highly conserved among various types of cells) that involved in the transport of cargos [47, 48]. To further verify the hypothesis mentioned above, Co-IP assays were implemented. HEK293T cells, expressing endogenous importins including importin α [48], were co-transfected with either of the following available plasmid combinations: pCMV9-3 × Flag-importinβ1/pICP22-EYFP, pcDNA-Flag-kα1 (importin α5)/pICP22-EYFP, pFLAG-CMV-transportin-1/pICP22-EYFP, Flag-kα2 (importin α1)/pICP22-EYFP, Flag-kα4 (importin α3)/pICP22-EYFP or Flag-kα6 (importin α7)/pICP22-EYFP. 24 h post transfection, cell lysates were collected and Co-IP was carried out using anti-Flag mAb or mouse IgG. Immunoprecipitated proteins were subjected to western-blot analysis with Flag and YFP antibodies. As results, P-ICP22 was efficiently co-immunoprecipitated with importin α1 (Fig. 5d) and α7 (Fig. 5f), but not importin β1 (Fig. 5a), importin α5 (Fig. 4b), transportin-1 (Fig. 5c) or importin α3 (Fig. 5e). As control, no target protein was immunoprecipitated by IgG (Fig. 5), indicating P-ICP22 is associated with importin α1 and α7.

In conclusion, the subcellular localization, localization signal and nuclear transport mechanism of P-ICP22 were identified in this study. P-ICP22 is shown to principally localized to the nucleus. By constructing a series of deletion mutants fused with EYFP and fluorescence microscopy analysis, we identified an N-terminal arginine-rich functional NLS (^41^PASTPTPPKR GRYVVEHPEY^60^) in P-ICP22, and ^49^KR^50^ is the crucial aa for its nuclear localization. In addition, P-ICP22 was characterized to import into the nucleus via Ran-, importin α1- and α7- mediated pathway, which would expand its list of the host targets, and P-ICP22 probably transports into the nucleus via Ran-, importin α1- and α7- mediated pathway to selectively modify the transcription of different kinetic classes of PRV genes. Therefore, this study reveals the molecular mechanisms for the nuclear transport of P-ICP22, and will advance the understanding of P-ICP22-mediated biological functions in PRV replication.

## Methods

### Plasmids construction

All enzymes used for cloning processes were purchased from Takara (Dalian, China) except DNA polymerase KOD FX from TOYOBO and T4 DNA ligase from New England Biolabs (MA, USA). PRV BAC pBecker2 was a generous gift from Dr. Lynn W. Enquist [49]. The P-ICP22 ORF (composed of 1047 bp) was amplified via PCR from the genomic DNA of PRV Becker strain pBecker2. After the PCR amplified product was validated as the intended product, it was purified using a PCR gel purification kit (Qiagen) according to the manufacturer’s instructions. The product was then digested with *EcoR*I and *BamH*I and inserted into the correspondingly digested green fluorescent protein variant mammalian expression vector pEYFP-N1 (Clontech) encoding EYFP at 16 °C overnight using T4 DNA ligase, to create the recombinant plasmid named pP-ICP22-EYFP, as described previously [3, 26, 50–53]. After mini-scale isolation of plasmid DNA using Qiagen plasmid Mini kits (Qiagen), the presence of the appropriate insert in the obtained plasmid was confirmed by PCR, restriction analysis and sequencing. Subsequently, the P-ICP22 ORF was subcloned into pEYFP-C1 (Clontech) to yield pEYFP-P-ICP22. The series of mutant deletions were generated by PCR-ligation-PCR mutagenesis [54] using appropriate primers and cloned into pEYFP-N1 as described above, all the N terminal deleted fusion proteins were inserted with an artificial ATG start codon starting at internal positions in P-ICP22 of the fusion constructs. Finally, all constructs were verified by PCR, restriction analysis and sequencing. Dominant negative (DN) mutant RanGTP [55], DN kα1 [42] and DN kβ1 [41] were subcloned into pECFP-N1 (Clontech) to generate pRan-Q69L-ECFP, pDN kα1-ECFP and pDN kβ1-ECFP, respectively. RFP-Bimax2 and RFP-M9M were generous gifts from Dr. Nobuyuki Nukina [56]. pcDNA-Flag-kα1 (importin α5), Flag-kα6 (importin α7) [57], Flag-kα2 (importin α1), Flag-kα4 (importin α3) [58] and pCMV9-3 × Flag-importinβ1 were the generous gifts from Drs. Yoshihiro Yoneda, Reinhard Depping and Ben Margolis, respectively. The transportin-1 ORF was amplified from pGEX-tev-kapbeta2 [59] and subcloned into pFLAG-CMV-2 (Sigma) to produce pFLAG-CMV-transportin-1. All the primers used in this study are available upon request.

### Transfection and fluorescence microscopy

Transfection and fluorescence microscopy assays were fulfilled as shown in our previous studies [23, 53]. Briefly, to express the proteins in vitro, COS-7 cells were plated onto six-well plates (Corning) in DMEM with 10 % FBS at a density of ~2.5 × 10^5^ cells per well overnight to 60–80 % confluency before transfection. The next day, monolayer of COS-7 cells were transfected with 1.0–1.5 μg of indicated plasmid DNA mixed with Thermo Scientific TurboFect Transfection Reagent following the manufacturer’s instructions. The transfection efficiency (~60 %) was determined by EYFP. 24 h post transfection, cells were washed with fresh growth medium and subjected to fluorescence microscopy with a Zeiss Axiovert 200 M microscope (Germany). In the same experiment, each transfection was performed for at least three times. Data shown were from one representative experiment. All the photomicrographs were taken under a magnification of 400×. Each photomicrograph represents an enormous majority of the cells with similar subcellular localization. Both fluorescent images of EYFP and ECFP fusion proteins were presented in pseudocolor, green and red, respectively, and the merged images were shown to prove the colocalization and appeared in yellow signals. All scale bars indicate 10 um. Images were processed by Adobe Photoshop.

### Co-IP and western-blot

Co-IP and western-blot were manipulated as described previously [23, 53]. Briefly, HEK293T cells (~2.5 × 10^6^) were co-transfected with 5 μg of each of the indicated expression plasmids bearing EYFP or Flag tag. Transfected cells were harvested 24 h post transfection and lysed on ice with 1 mL of lysis buffer. For each IP, a 0.5 mL aliquot of lysate was incubated with 0.5 μg of the anti-Flag monoclonal antibody (mAb) or nonspecific control mouse antibody (IgG) and 30 μL of 1:1 slurry of Protein A/G PLUS-Agarose (Santa Cruz Biotechnology) for at least 4 h or overnight at 4 °C. The Sepharose beads were washed three times with 1 mL of lysis buffer containing 500 mM NaCl. Subsequently, the immunoprecipitated proteins, as well as the cell lysates, were subjected to immunoblotting (IB) analysis with anti-Flag mAb (Sigma) and anti-YFP polyclonal antibody (pAb) (Santa Cruz Biotechnology). All Co-IP were repeated at least two times, and similar data were obtained.

## Abbreviations

PRV: pseudorabies virus
P-ICP22: PRV ICP22
IE: immediate-early
E: early
L: late
VZV: varicella-zoster virus
HSV-1: herpes simplex virus 1
BHV-1: bovine herpesvirus type 1
EYFP: enhanced yellow fluorescent protein
NLS: nuclear localization signal
DN: dominant negative
ECFP: enhanced cyan fluorescent protein
Co-IP: co-immunoprecipitation
mAb: monoclonal antibody
pAb: polyclonal antibody
IB: immunoblotting
